# Draft Genome Sequence of the Tyramine Producer *Enterococcus durans* Strain IPLA 655

**DOI:** 10.1128/genomeA.00265-13

**Published:** 2013-05-16

**Authors:** Victor Ladero, Daniel M. Linares, Beatriz del Rio, Maria Fernandez, M. Cruz Martin, Miguel A. Alvarez

**Affiliations:** Instituto de Productos Lácteos de Asturias, IPLA-CSIC, Paseo Rio Linares s/n, Villaviciosa, Asturias, Spain

## Abstract

We here report a 3.059-Mbp draft assembly for the genome of *Enterococcus durans* strain IPLA 655. This dairy isolate provides a model for studying the regulation of the biosynthesis of tyramine (a toxic compound). These results should aid our understanding of tyramine production and allow tyramine accumulation in food to be reduced.

## GENOME ANNOUNCEMENT

*Enterococcus durans* is a minor component of the intestinal microbiota of humans and domestic animals (1). It is commonly found in animal-derived food products, especially milk and cheese (2). This bacterium produces tyramine—a biogenic amine (BA) frequently present in fermented foods—by decarboxylating tyrosine. The ingestion of food with high tyramine concentrations causes an intoxication, known as the cheese reaction, characterized by migraine, hypertension, etc. (3). Tyramine production in bacteria has been related to energy production and the acidic stress response (4).

*E*. *durans* strain IPLA 655, which was isolated from a traditional cheese (5), provides a model for research into tyramine production (6–9). A genomic library of 0.5 kbp was constructed and subjected to paired-end sequencing (providing approximately 150-fold coverage) using a HiSeq 1000 System sequencer (Illumina) at the Beijing Genomics Institute (China). Quality-filtered reads were assembled into contigs using Velvet software (http://www.ebi.ac.uk/~zerbino/velvet/). Annotation was performed using the Prokaryotic Genomes Annotation Pipeline (PGAAP) at the NCBI (http://www.ncbi.nlm.nih.gov/genomes/static/Pipeline.html), and improved with results obtained from BLAST analysis (http://blast.ncbi.nlm.nih.gov). The draft genome sequence of *E. durans* IPLA 655 involves 3,059,052 bp, a GC content of 37.7%, and 141 contigs ranging from 201 to 203,388 bp. It encodes 2,853 predicted coding sequences. Single predicted copies of 16S, 23S, and 5S rRNA genes were found, as well as 51 genes for tRNAs.

In agreement with its dairy origin, *E. durans* IPLA 655 may metabolize lactose; a lactose phosphotransferase operon (*lacXGEFDCBAR*) was identified in its genome. However, no extracellular caseinolytic protease genes were found. The genome contains the tyramine production cluster, but no other BA clusters.

BA production clusters generally include a gene similar to those encoding the corresponding aminoacyl-tRNA synthetase, but with unproven function. Surprisingly, analysis of the *E. durans* IPLA 655 genome showed no other tyrosyl-tRNA synthetase gene besides the tyrosyl-tRNA synthetase-like gene (*tyrS*) in the tyramine cluster. This confirms that *tyrS* encodes a tyrosyl-tRNA synthetase. Upregulated at a low pH (9), when tyrosine is decarboxylated to counteract acidic stress, the increased product would ensure the supply of tyrosine for protein biosynthesis.

No evidence of virulence-related genes or antibiotic resistance genes was seen. At least three prophages were detected; these were most homologous with prophages of *E. faecium*. Some genes involved in plasmid mobilization or replication were detected, along with two putative toxin/anti-toxin plasmid stabilization systems, in agreement with the presence of at least three plasmids (data not shown).

The genome sequence of *E. durans* IPLA 655 should aid our understanding of the genetic regulation and physiological significance of tyramine biosynthesis, and perhaps suggest ways of reducing tyramine accumulation in food.

### Nucleotide sequence accession numbers.

The results of this whole-genome shotgun project were deposited in the DDBJ/EMBL/GenBank database under accession number AOSM00000000. The version of the genome described here has the accession number AOSM01000000.
